# Exclusive breastfeeding among working mothers in Kenya: Perspectives from women, families and employers

**DOI:** 10.1111/mcn.13194

**Published:** 2021-05-05

**Authors:** Scott B. Ickes, Hannah Sanders, Donna M. Denno, Jennifer A. Myhre, Joyceline Kinyua, Benson Singa, Hellen Sankaine Lemein, Lora L. Iannotti, Carey Farquhar, Judd L. Walson, Ruth Nduati

**Affiliations:** ^1^ Department of Applied Health Science Wheaton College Wheaton Illinois USA; ^2^ Department of Health Services University of Washington Seattle Washington USA; ^3^ Department of Global Health University of Washington Seattle Washington USA; ^4^ Kenya Medical Research Institute Nairobi Kenya; ^5^ Department of Pediatrics University of Washington Seattle Washington USA; ^6^ Childhood Acute Illnesses Network (CHAIN) Nairobi Kenya; ^7^ Naivasha Sub‐County Referral Hospital, Serge East Africa Naivasha Kenya; ^8^ Brown School Washington University in St. Louis St. Louis Missouri USA; ^9^ Departments of Medicine (Allergy and Infectious Disease) and Epidemiology University of Washington Seattle Washington USA; ^10^ Department of Pediatrics and Child Health University of Nairobi Nairobi Kenya

**Keywords:** breastfeeding, caregiving, lactation, low‐income countries, policy, qualitative methods, social factors

## Abstract

Exclusive breastfeeding (EBF) for the first 6 months of life improves survival, growth and development. In Kenya, recent legislation and policies advocate for maternity leave and workplace support for breastfeeding and breast milk expression. We conducted a qualitative study to describe factors influencing EBF for 6 months among mothers employed in commercial agriculture and tourism. We interviewed employed mothers (*n* = 42), alternate caregivers and employed mothers' husbands (*n* = 20), healthcare providers (*n* = 21), daycare directors (*n* = 22) and commercial flower farm and hotel managers (*n* = 16) in Naivasha, Kenya. Despite recognizing the recommended duration for EBF, employed mothers describe the early cessation of EBF in preparation for their return to work. Managers reported supporting mothers through flexible work hours and duties. Yet, few workplaces have lactation spaces, and most considered adjusting schedules more feasible than breastfeeding during work. Managers and healthcare providers believed milk expression could prolong EBF but thought mothers lack experience with pumping. The most frequently suggested interventions for improving EBF duration were to expand schedule flexibility (100% of groups), provide on‐site daycare (80% of groups) and workplace lactation rooms (60% of groups), improve milk expression education and increase maternity leave length (60% of groups). Returning to work corresponds with numerous challenges including lack of proximate or on‐site childcare and low support for and experience with milk expression. These factors currently make EBF for 6 months unattainable for most mothers in these industries. Interventions and supports to improve breastfeeding upon return to work are recommended to strengthen employed mothers' opportunity for EBF.

**Abbreviations**
EBF: exclusive breastfeeding
LMICs: low‐and‐middle‐income countries

**Key messages**

- Despite recognizing the recommended duration for EBF, employed mothers describe the early cessation of EBF in preparation for their return to work.
- Managers reported supporting mothers through flexible work hours and duties. Yet, few workplaces have lactation spaces, and most considered adjusting schedules more feasible than breastfeeding during work.
- The most frequently suggested interventions for improving EBF duration were to expand schedule flexibility, provide on‐site daycare and workplace lactation rooms, improve milk expression education and increase maternity leave length.

## 1 INTRODUCTION

Exclusive breastfeeding (EBF) for 6 months is a critical health behaviour to reduce infant mortality and support health and cognitive development by preventing infections (Lee et al., 2016; Neovita, 2016; Victora et al., 2016). Despite efforts to improve BF practices globally, only 42% of infants are breastfed exclusively through 6 months. Most countries are not on track to achieve the global target of 50% (2020 Global Nutrition Report, 2020; Victora et al., 2016). Although reported EBF prevalence among Kenyan four to 5‐month‐olds increased from 2.7% in 2000 to 42% in 2014 (2020 Global Nutrition Report, 2020), Kenya's EBF duration is one of the shortest in the East Africa region at 3.3 months (ORC Macro, 2016).

Over the past two decades, female workforce participation in low‐ and middle‐income countries (LMICs) has increased, along with a shift from informal to more formalized employment (Adair et al., 2002; Head et al., 2014). Initiatives such as the Millennium Development Goals have promoted women's work, improve health and alleviate poverty in LMICs (Kabiru et al., 2018). Given its growing gross domestic product (GDP) and high female workforce participation (World Bank, 2019), Kenya is an important setting to understand maternal employment's influence on child health outcomes. In Kenya, 62.4% of women are employed in some capacity, comprising 49.4% of the national labour force (International Labour Organization (ILO), 2017, 2019).

The Naivasha region is home to a large commercial agriculture industry with many multinational farms that export commodities such as tea, coffee and flowers (Kabiru et al., 2018). Growth in export values and volumes of cut flowers has made the floriculture industry Kenya's largest employer, directly supplying jobs to over 100,000 people of which over two‐thirds are women (Kabiru et al., 2018; Kenya Flower Council, 2020). Additionally, Kenya's tourism industry employs millions of women in hotels and resorts. In Naivasha, a lake and several national parks provide tourism‐related employment (Sanghi et al., 2017). The high female employment rate in floriculture and tourism makes these industries important child health influencers.

Multilevel—individual, community and societal—factors influence EBF practices, including legislation, health services, workplace support policies and services, as well as social norms (Bhandari et al., 2008; Rollins et al., 2016). In LMICs, employed women are less likely to maintain EBF for the recommended 6 months (Oddo & Ickes, 2018). Early EBF cessation has been associated with workplace factors, including short maternity leave, full‐time employment, not pumping breast milk, lacking a lactation break, workplace distance from children and inflexible work schedules (Kebede et al., 2020). Kenyan law requires all employers provide BF supports, including paid 3‐month maternity leave, private BF rooms, refrigeration for expressed milk and flexible work schedules (Government of Kenya, 2017; National Council for Law Reporting, 2012). The Kenya Ministry of Health developed guidance to facilitate the implementation of these policies (Kenya Ministry of Health, 2018). However, despite these policies and guidelines, formal employment in Naivasha is associated with a 36% lower prevalence of EBF at 14 weeks compared with informal or no employment (Ickes et al., 2021).

As workplaces adapt to new BF policy requirements, it is essential to understand the factors that influence policy adherence, implementation and female employees' willingness to use these workplace supports. The purpose of this study is to understand the barriers and facilitating factors on the capacity to maintain EBF for the recommended 6‐month duration among women employed in the commercial agriculture and tourism industries.

## 2 METHODS

### 2.1 Overview

We conducted a qualitative study in Naivasha, Kenya, using semi‐structured, in‐depth, key informant interviews (KIIs) among five participant groups. We recruited mothers, alternative caregivers, healthcare providers, childcare centre directors and commercial flower farm and hotel managers. A grounded theory approach, with intentionally open‐ended research questions, guided our study. Participant sampling was driven by data saturation, and interview questions were adapted to explore emergent findings through an inductive process (Foley & Timonen, 2015; Glaser & Strauss, 1967).

### 2.2 Study setting

Naivasha is a peri‐urban city in Nakuru County, located 100 km north of Nairobi, with approximately 170,000 people (Kenya National Bureau of Statistics [KNBS], 2019). This area supports the largest concentration of commercial flower farms in Kenya and is the primary employment source in this region. Most floriculture employees reside in densely populated peri‐urban informal settlements, with variable access to electricity and sanitation services.

### 2.3 Data collection tools

We developed preliminary interview guides from a review of evidence and the Lancet series conceptual framework on employment and BF (Bhandari et al., 2008; Rollins et al., 2016), analysis of national policies, and through study personnel observation of child health immunization days, childcare centres in the community and based at farms and commercial farms. The research team pilot tested the guides with each participant group to providing input about the data quality and richness. We iteratively revised guides during the study to pursue emergent areas of exploration and further improve the ability to obtain rich responses.

### 2.4 Training of interviewers

Three members of the research team conducted the interviews (SI, HL, HS). The team leader led training on qualitative interviewing, which included demonstration interviews. The training emphasized research ethics, open‐ended questions, active listening, cultural and power dimensions of interviews, establishing rapport and emotional regulation during interviews (McGrath et al., 2019).

Members of the research team had pre‐existing professional relationships with a few healthcare providers but no prior relationships with any other participants. A translator with Kiswahili as their first language translated all study guides. A third‐party review board evaluated and certified translation accuracy.

### 2.5 Study participants and recruitment

A recent conceptual framework outlining the interrelated childcare, workplace, health system and household influences of BF practices informed the recruitment strategy (Rollins et al., 2016). We recruited mothers from maternity wards and immunization clinics at three health centres: Naivasha District Hospital, Karagita Dispensary and South Lake Medical Center. After explaining the nature of the interview to mothers, we invited participation. Mothers were eligible if they were presently employed (including during maternity leave) at a commercial flower farm or hotel and had a child younger than 12 months. Alternate caregivers (grandmothers and aunts) and fathers were recruited either when they accompanied mothers to the delivery wards, immunization clinics or by phone when mothers provided their phone numbers. We scheduled all in‐person interviews within two days of recruitment. All fathers and alternate caregivers were related to a mother who was interviewed.

Healthcare providers, including doctors, nurses, nutritionists and community health workers, were recruited primarily at Naivasha District Hospital. Additionally, we sought healthcare providers at farm clinics and dispensaries through in‐person visits and snowball sampling. Daycare directors were identified through informants from three primary communities where mothers who work at commercial farms and hotels reside: Karagita, Kihoto and Naivasha towns. Daycares were visited in person, and directors were informed of the study purpose and invited to participate. Farm and hotel managers were recruited via email and phone using contact information obtained from company websites and through dropping letters at main offices by hand. We scheduled all meetings via phone calls and conducted all interviews in person. We sought additional managers' contact information using snowball sampling. We obtained written informed consent for all participants. All participants except farm and hotel managers received a soap as a token of appreciation for participating.

### 2.6 Data collection and analysis

Field notes were taken by the research team and were used to contextualize the analysis. Interviews lasted approximately 30–60 min. Transcripts were not returned to the participants. Healthcare providers were invited to presentations of the research findings at the continuing medical education series at the Naivasha Sub‐County District Hospital.

Interviews were audio‐recorded and translated to English before transcription, when applicable. Translations were checked for accuracy by a third‐party reviewer. Field notes were also taken and used to contextualize the analysis. Transcripts were double coded using Dedoose software. Rich text excerpts with de‐identified demographic information (participant group and interview number) were initially coded into one or more of 37 codes developed throughout the analysis process. We applied the framework method (Gale et al., 2013) to aggregate these codes into nine families further using a node tree: (1) current BF practices; (2) workplace supports implementation challenges; (3) employer characteristics and supports; (4) workplace barriers; (5) workplace opportunities; (6) healthcare promotion practices; (7) BF in the context of HIV; (8) childcare challenges and opportunities; and (9) desired changes from mothers. Using a constant comparative method and a coding matrix organized by these nine families, we identified themes and subthemes (STs) from participant responses (Miles et al., 2020). Within and across participants groups, divergent responses were included to represent opposing perspectives related to the main findings. Participant groups were analysed simultaneously to identify how and where different key informant responses differed or concurred. Four members of the team double coded each transcript. Participants did not receive transcript copies nor provide feedback on the findings. Healthcare providers were invited to a presentation on the research findings at Naivasha Sub‐County District Hospital's continuing medical education series.

We compared participant group's recommended interventions with the evidence base for these recommendations using a search of PubMed for peer‐reviewed literature. We ordered responses by identifying those suggested by the highest number of participant groups. A minimum of one participant recommending an intervention was required to attribute the recommendation to the group.

### 2.7 Ethical considerations

The study protocol followed the Consolidated Criteria for Reporting Qualitative Research (COREQ) (Table S1) (Tong et al., 2007). The Kenya Medical Research Institute and Wheaton College Institutional Review Boards approved all study procedures.

## 3 RESULTS

### 3.1 Participant sample

We conducted 121 KII across five participant groups. Table 1 summarizes the respective sample sizes, interview locations, participation rates and information sought from each group. Forty‐two mothers employed in a flower farm or hotel, 20 fathers and alternate caregivers, 21 healthcare providers, 22 daycare directors, 16 farm and hotel managers were interviewed. The participation rate was 100% among mothers and healthcare providers, 95% among fathers and alternative caregivers, 88% among daycare directors and 55% among farm and hotel managers. The main reason for refusal from managers was reported lack of time to participate or interest in the study.

**TABLE 1:** Participant groups, sample size, interview setting and information sought from in‐depth interview participants

Participant group	Sample size and interview setting	Participation rate	Purpose/key information sought in in‐depth interviews
Mothers employed at commercial flower farms or hotels	42 28 employed at farms 14 employed at hotels Immunization clinic and participant homes	100% (42 of 42)	To identify awareness of existing workplace policies; barriers to use of maternal leave policies and breastfeeding (BF)‐support practices; feasibility of expressing and storing milk at work.
Fathers and alternate caregivers	20 10 fathers 10 grandmothers and aunts Participant homes or health centres	95% (20 of 21)	Breastfeeding beliefs; experience with supporting/influencing mother's breastfeeding.
Healthcare providers (delivery ward and maternal and child health nurses, obstetricians, nutritionists and community health workers)	21 8 nurses 3 obstetricians 3 paediatricians 4 nutritionists 3 community health workers Workplace, public and private health centres	100% (21 of 21)	Challenges to support exclusive breastfeeding in the first month of life; identify current supportive efforts and hospital policies to support BF among mothers after delivery.
Daycare centre directors	22 Director home or daycare centre	88% (22 of 25)	Feasibility of storing expressed milk; challenges to support nursing mothers; role of childcare centre directors in supporting BF and opportunities for additional support.
Managers at commercial flower farms and hotels	16 workplace, farm or hotel	55% (16 of 29)	Successful implementation of workplace policies; factors that prevent farms from adopting policies to support BF; assessment of contextual factors (facilities, lactation breaks, refrigeration, on‐site daycare).

Among the mothers, the median (SE) age was 29.7 (1.1) years. The mean (SE) age of their youngest child was 4.5 (0.6) months, and most (90%) were married. Mothers were employed at eight commercial flower farms and four hotels. One owned a breast milk pump. Among mothers with a child under 6 months (*n* = 21), one (5%) had stopped BF, most (*n* = 13, 62%) were currently practicing mixed feeding and seven (33%) reported EBF.

Data from the coding families were summarized into five themes related to (1) employment‐related EBF challenges; (2) experiences with breast milk expression; (3) BF in the context of HIV; (4) workplace support for nursing mothers; and (5) recommended interventions to support EBF.

Table 2 reports each participant group's perspectives, using quotes combined under key themes and subthemes (STs). Table S2 reports expanded participants responses.

**TABLE 2:** Key themes and subthemes from key informant interviews

Theme 1: Multiple participant groups identified formal employment as a challenge to practicing EBF for the recommended 6‐month duration.
Subtheme #1.1: Despite recognizing the recommended 6‐month duration for EBF and health benefits such as healthy growth and improved immunity, employed mothers reported beginning mixed feeding in preparation for their return to work after their 3‐month maternity leave.
Subtheme #1.2: Healthcare providers reported providing evidence‐based breastfeeding education to mothers but concurred that maternal employment is a challenge to practicing EBF for 6 months.
Theme 2: Though managers and healthcare providers recognized pumped milk as an opportunity for working mothers to continue to practice EBF, few employed mothers reported expressing breast milk.
Subtheme #2.1: Managers, healthcare workers and daycare directors all concurred that milk expression might be challenging for or undesirable to mothers. They voiced doubts that mothers would readily adapt to expressing and storing milk.
Subtheme #2.2: Most mothers had heard of milk expression, and many mothers expressed an interest in pumping to extend EBF after returning to work. However, they did not feel expression was feasible due inadequate instruction and demonstration, lack of hygienic environment and equipment (i.e., breast pumps, bottles and refrigeration).
Subtheme #2.3: Daycare directors suggested that mothers are hesitant to leave expressed milk to feed their children during the workday because they lack refrigeration at their daycares to store milk.
Subtheme #2.4: Lack of trust in providers negatively influences mothers' willingness to leave expressed milk at daycares.
Theme 3: Healthcare providers reported that though they provide evidence‐based breastfeeding education consistent with global guidance on infant feeding and HIV, employed mothers are challenged to practice EBF through 6 months.
Subtheme #3.1: HIV‐infected mothers were often enrolled in HIV care services, which included education on infant feeding. Despite these additional supports, providers observed that work poses a challenge for some mothers to practice EBF through 6 months .
Subtheme #3.2: Some HIV‐infected mothers reported additional benefits due to their status, such as extended maternity leaves and transportation to return home during the workday to breastfeed. Other HIV‐infected mothers indicated that they could only manage to practice EBF through 3 months when the return to work would force them to begin mixed feeding or wean from mother's milk if EBF was no longer an option.
Theme 4: Workplaces recognized the need to support breastfeeding and identify some practical employee benefits; however, employers describe maternal‐level limitations to using workplace benefits.
Subtheme #4.1: Most farm managers reported that mothers prefer to arrive later or leave earlier rather than visiting children during the workday for feeding.
Subtheme #4.2: Few farms or hotels reported having designated lactation areas at the time of the study, and several workplace managers described plans to comply with new national policy.
Subtheme #4.3: Managers described that cultural factors may prevent mothers from using private lactation rooms at workplaces once established. Healthcare providers also noted that mothers may lack knowledge of and experience with expressing and storing milk.
Theme 5: Despite significant challenges, opportunities to improve support for EBF exist in household, community, workplace and health system settings.
Subtheme #5.1: Though mothers offered various suggestions to improve the feasibility of continuing EBF, including flexible hours and lactation support, many preferred the addition of workplace daycare.
Subtheme #5.2: Fathers and alternate caregivers described several changes that would improve the feasibility of EBF for working mothers. The most frequent suggestion from this group was a more substantial reduction and flexibility of work hours upon returning to work.
Subtheme #5.3 Healthcare providers most frequently report improvement of perinatal and community education to support the continuation of EBF, and recommend on‐site daycares, supplies to support milk expression and governmental policy changes to maternity leave and lactation rooms.
Subtheme #5.4: Daycare directors were in consensus that mothers need both education and the provision of supplies such as pumps, bottles and refrigerators to support breast milk expression as a means to facilitate EBF upon return to work.
Subtheme #5.5: Most employers perceived they are providing enough support for lactating mothers through the current maternity leave duration, flexibility in hours and, in a few cases, on‐site daycare. Those who did not have on‐site daycares felt it could be helpful in supporting EBF.

Abbreviation: EBF, exclusive breastfeeding.

#### 3.1.1 Theme 1: Employment‐related challenges to EBF

Multiple participant groups identified formal employment as a challenge to practicing EBF for the recommended 6‐month duration. Mothers described that work poses numerous obstacles to practicing EBF after returning from maternity leave: inability to travel home or to daycare to breastfeed during the workday, lack of knowledge about and experience with milk expression and storage and lack of BF spaces and pumping equipment.

Despite recognizing the recommended 6‐month duration for EBF and health benefits such as healthy growth and improved immunity, employed mothers reported beginning mixed feeding in preparation for their return to work after their 3‐month maternity leave (ST 1.1). Though employed mothers may ‘wish to exclusively breastfeed’ through 6 months, they perceived that going ‘back to work’ necessitates the early introduction of other foods (Theme 1, ST 1.1, Daycare #9).

Mothers reasoned that early introduction of foods such as porridge and cow's milk would help their child adjust to a new diet before they return to full‐time work, ‘I started to feed by the end of maternity leave, the third month. I started him with milk and porridge lightly’ (Theme 1, ST 1.1, Mother #30). And, ‘For me, I have to give food before I report back to work so that by the time I go to work, the baby will be used to feeding on other foods’ (Theme 1, ST 1.1, Mother #20).

Breastfeeding education and promotion, provided as individual and group counselling, are recommended during antenatal and postnatal care. These services were reported by healthcare providers and mothers to be provided through the health system. Most mothers and some fathers reported receiving perinatal BF education at hospitals or clinics. Some commercial farms have on‐site health clinics that offer BF promotion during antenatal visits. The authors have observed in multiple clinics that BF education shifts from individualized counselling, which begins during pregnancy and immediately postpartum to group education sessions during maternal and child health clinic visits.

Although mothers desire to feed according to ideal practices, they described the challenge of doing so*:* ‘I hear one is supposed to breastfeed for six months before weaning. Although, for me, I cannot manage because of work’ (Theme 1, ST 1.2, Mother #20). Many healthcare providers concurred, ‘Mostly, those mothers fail to do exclusive BF because of work, and especially those who are working in the flower farms who go very early in the morning and coming in the evening’ (Theme 1, ST 1.2, Healthcare #8). Another healthcare worker reported that during education sessions, mothers questioned EBF feasibility upon returning to work:

They know because we are trying to teach them, trying to educate them. But still, when you educate them, they tell you, ‘Where will I get that time?’ Cause they get up early, they go to work early. So, no. They don't have that enough time to do exclusive breastfeeding (Theme 1, ST 1.2, Healthcare #18).

#### 3.1.2 Theme 2: Experiences with breast milk expression

Though managers and healthcare providers recognized pumped milk as an opportunity for working mothers to continue EBF, few employed mothers reported expressing breast milk. Managers, healthcare providers and daycare directors all concurred that milk expression might be challenging because of limited knowledge and experience with expression and milk storage (ST 2.1). They also felt that mothers may find the milk expression undesirable and may also experience adverse reactions from family and peers because ‘culturally, it is not very acceptable apart from those who have been a bit modernized’ (Theme 2, ST 2.1, Hotel #1). Healthcare providers also felt that ‘[mothers] don't have enough education’ and had concerns about insufficient supply and fear of spoilage as potential barriers (Theme 2, ST 2.1, Healthcare #15).

Many mothers had heard of this practice from healthcare providers, observed mothers of premature babies, or had seen pumps for sale at stores. Despite beliefs among some daycare directors that mothers ‘have no interest’ in milk expression, many mothers described a desire to pump as a means to extend EBF after returning to work (Theme 2, ST 2.2, Daycare #2). However, they did not feel this was a feasible option due to lack of adequate instruction and demonstration on how to do so and lack of a hygienic environment and necessary equipment (i.e., breast pumps and bottles and refrigeration for safe storage) (ST 2.2).

I have heard about [pumping milk], but I can't do it because I don't have the items to pump. I hear you must keep the milk in a clean place, and also, the person feeding the baby must check the hygiene (Theme 2, ST 2.2 Mother #31).

Most mothers indicated that on‐site lactation rooms would enable expression and storage at their workplaces. However, concern over pesticide exposure (e.g., ‘there are so many chemicals at the farm’ [Theme 2, ST 2.2, Mother #32]) was a deterrent for some.

Daycare directors suggested that mothers are hesitant to leave expressed milk for their children because the daycares lack refrigeration to store milk (ST 2.3). Though healthcare providers reported attempts to explain that if expressed milk ‘stays in a clean place for less than eight hours,’ refrigeration is unnecessary (Theme 2, ST 2.3, Healthcare #15), mothers and daycare directors reported scepticism about unrefrigerated milk storage safety:

[EBF] has not been very successful. Some of [the mothers] were not very satisfied. But it is because we don't have the facilities. I know that we would need a fridge for that baby. The milk can continually be fresh, and so we don't have that. We don't have power (electricity). So, we are limited (Theme 2, ST 2.3, Daycare #2).

Furthermore, there was evidence that some mothers attempt to leave expressed milk but became frustrated and discontinued shortly after returning to work:

I have seen them; there is a trend. Immediately when [breastfeeding mothers] go back to work, they really try…The first day they will bring like three‐quarters of a bottle of expressed breastmilk, and then they go to work. They stay in hot conditions, don't feed, and have all of the stress of working. And by the end of that week, the mother will not provide even a little amount of milk. So, they just stop. But they try the first week (Theme 2, ST 2.3, Daycare #3).

Lack of trust and a concern that many daycares are ‘not registered’ and have high child‐to‐provider ratios negatively influence mothers' willingness to leave expressed milk at daycares (Theme 2, ST 2.4, Healthcare #17). Mothers are unsure that their milk is appropriately stored and reheated and that their child ‘will be fed the same milk (Theme 2, ST 2.4, Mother #6)’ that she left for her child and not the milk from another mother.

#### 3.1.3 Theme 3: BF in the context of HIV

Healthcare providers reported that they counsel HIV‐infected mothers on infant feeding strategies to prevent mother‐to‐child transmission. Some providers described their BF promotion efforts for HIV‐infected mothers to be more in‐depth due to the added importance of preventing vertical transmission, which was perceived to be low. HIV‐infected mothers were often enrolled in HIV care services, which also include infant feeding education. Despite these supports, providers observed that work poses a challenge for some mothers to practice EBF through 6 months (ST 3.1). While healthcare providers noted that HIV‐infected mothers might be ‘more careful’ to maintain EBF before returning to work (Theme 3, ST 3.1, Healthcare #3), they concluded that HIV‐infected mothers are generally unable to express milk.

Initially, they were very resistant. There was the perception that HIV positive mothers should not breastfeed their babies. But nowadays, they are very comfortable with it. They take it positively, and they follow the rules that we are giving them. Exclusive breastfeeding is a must. But at some point, we get challenges, like a mother says she only has three months' maternity leave, so after three months, she should be going back to work. So how will the baby be managed? So, we tell her she should pump; she should express the milk. And we tell her that breastmilk takes eight hours before spoiling. But most of them don't take it positively because they say they don't have enough milk. And how will the caregiver handle the baby and clean? And maybe there is still fear about the stigma (Theme 3, ST 3.1, Healthcare #7).

In some interviews, mothers voluntarily disclosed their HIV status. Other HIV‐infected mothers indicated that they could only manage EBF through 3 months. After that leave, the return to work forces them to begin mixed feeding or stop BF altogether due to concerns about mixed feeding (ST 3.2)*,* ‘[When I return to work] I will have to stop breastfeeding completely because of my HIV status’ (Mother #16).

#### 3.1.4 Theme 4: Workplace supports for working mothers

Workplace managers recognized the need to support BF and listed some BF‐related benefits that they provide; however, they described maternal‐level barriers to their use. In compliance with the 2017 Kenya Bill of Health, both farm and hotel managers reported supporting lactating mothers through flexible work schedules and re‐assignment to less physically demanding roles (e.g., ‘lighter duties’). Most farm managers said mothers prefer to arrive later or leave earlier rather than visit children during the workday for feeding,

I think what we are doing is perfect because it is very flexible for us. We have the arrangements for those who probably want to work late, work normal hours, or combine their lunch hour with their tea break, so they leave much earlier. Because we give one hour for lunch, forty minutes for a break. So, when they report at around seven‐thirty, some leave at midday (Theme 4, ST 4.1, Farm #1).

Hotel managers described that the hospitality industry offers more flexibility by its nature because there are multiple shifts per day; however, guest demands take precedent and may hinder employee's breaks and delay departure from work.

We can also give what we call a ‘broken shift.’ Broken shifts mean that because most of the people usually reside not very far from the hotel, maybe in Karagita, you know, in Naivasha town… [The] broken shift means that you can work for four, or just … three hours, then you take one hour, go home, feed the kid, and then you can come back (Hotel #2).

Many farms located far from residential areas provide separate transportation for lactating mothers to accommodate a late start or early departure. However, the distance between work and childcare, combined with lack of transportation, inhibits mothers from travelling to breastfeed during lunch breaks. Some farms, especially those with employee residences, also have subsidized on‐site daycare that allows mothers to breastfeed during shifts. Hotels did not report offering daycares or on‐site housing.

Employers also reported providing paid time off for mothers whose children became ill.

Few farm or hotel managers reported having designated lactation areas at the time of the study, noting this was a ‘new law,’ and most employers have not been able to ‘implement this so far’ (Theme 4, ST 4.2, Manager #2). Several managers described plans to comply with the new national policy (ST 4.2).

This year, a new labor law came active, and you have to have a breastfeeding room and we … said to ourselves, ‘Are we just building a room, and we put a signboard on the door, and that is it?’ Or are we actually looking into ‘How can we help make it into a success?’ We are looking into now is if we could get a hospital‐grade pumping with removable cups and a fridge that we can actually have it here so our employees can actually use it. Maybe five people at the same time, because at the moment, I think we will have five breastfeeding ladies at the same time (Theme 4, ST 4.2, Farm #6).

The farms that reported having lactation rooms noted these are for those in administrative positions with more hygienic offices and a ‘higher level of understanding’ but not for hourly ‘workers’ situated in unclean areas such as greenhouses or packing facilities (Theme 4, ST 4.2, Farm #4). Mangers felt that these mothers would need to change clothing or wash before BF, which they perceived was not currently feasible in their settings.

Healthcare providers also noted that mothers might lack knowledge of and experience expressing and storing milk (ST 4.3). Managers queried whether private lactation rooms might go unused due to cultural factors:

If I put [a lactation room] here, then nobody is going to use it because it is not… accepted yet. I don't know how it is in Nairobi and the higher educated sectors, but I think that will be the biggest challenge to actually having people understand the right thing to do (Theme 4, ST 4.3, Farm #8).

Mothers did not concur with farm manager perceptions in all cases, indicating that they would be willing to express breast milk for their baby if they were provided breast milk instruction, a place to store the milk, a breast milk pump and a private lactation room. For some mothers, storage equipment may be just as critical as a lactation space. When asked if there would be ‘any issue with expressing at work,’ one mother replied: ‘No. If I had my own bottles and, for example, a pump, I would just express at the bathrooms. No one will be bothered’ (Theme 4, ST 4.3, Mother #37).

#### 3.1.5 Theme 5: Recommended interventions to support EBF

Despite significant challenges, opportunities to improve EBF support exist in households, communities, workplaces and health system settings. Though mothers offered various suggestions to enhance the feasibility of continued EBF, including flexible hours, many recommended workplace daycares, noting the current insufficiency of flexible work schedules:

[We need] a baby care at the farm so we can take our children there. So, whenever we are needed to breastfeed or be given breaks to breastfeed during the day. That will be of great help other than the one hour they give. Because getting transport home takes a while, there is not much difference with the other employees (Theme 5, ST 5.1, Mother #37).

Others also desired more time to breastfeed and transportation to visit their child during lunch breaks, indicating that mothers preferred to directly breastfeed children rather than expressing milk for feeding by a childcare provider or family member.

Fathers and alternate caregivers described several changes that would improve EBF feasibility for working mothers. Their most frequent suggestion was a more substantial reduction and flexibility of work hours upon returning to work (ST 5.2). However, this solution seemed second best to several fathers who believed the ideal would be to ‘extend [maternity leave] to six months’ (Theme 5, ST 5.2, Father #1).

Some companies may say we gave three‐month maternity leave. But three months … it's not six months, but the mother must continue breastfeeding after six months. It could be a sacrifice to provide that room or time for the mother to breastfeed at least three times a day, and she should go to work at least one hour late and that one hour and 30‐minute lunch break to travel. If they are from far, at least provide a room. It should not be looked at as a disadvantage of one hour, but as an advantage of building a next employee in the future, and if you don't do it, you will risk having an employee who is always sick (Theme 5, ST 5.2, Father #7).

Additionally, husbands reported supporting their partners' nutrition—by buying extra cow's milk or porridge so they ‘have enough breastmilk to breastfeed’ (Theme 5, ST 5.2, Father #3). Some fathers reported helping with cooking, laundry, changing diapers and collecting water to lighten their partners' housework burden.

Healthcare providers most frequently prioritized enhanced perinatal and community education to support EBF continuation through behaviour change.

Education is power. If you tell [these mothers] that breastmilk takes eight hours to get spoiled, they will understand and practice it. And telling them that doing exclusive breastfeeding will boost the immunity of this child, they will do it… When they have not yet delivered, they should be educated. In the community, we have Community Health Workers … they should be educating the mothers. When the mother delivers here, we always give health education, and we insist on breastfeeding, that exclusive breastfeeding. (Theme 5, ST 5.3, Healthcare #7).

Healthcare providers also recommended on‐site daycares, supplies to support milk expression and governmental policies lengthen maternity leave and mandate lactation rooms (ST 5.3). They recognized the need for multisectoral action, including ‘empowering the caregivers,’ ‘linking with the community … like churches or other schools’ and ‘giving the mothers or companies enough encouragement’ in EBF promotion (Theme 5, ST 5.3, Healthcare #3, #4 and #11).

Daycare directors concurred that both education and provision of breastmilk expression supplies (e.g., pumps, bottles, refrigerators) would facilitate EBF upon return to work (ST 5.4). Additionally, they felt that daycare workers should receive training on breast milk expression by healthcare providers, so they ‘have facts to tell the mothers’ (Theme 5, ST 5.4, Daycare #21). Daycare directors perceived such training breast milk ‘would help because people believe doctors a lot, and nurses’ and saw the daycare's role as ‘enforcing’ what mothers learned (Theme 5, ST 5.4, Daycare #2).

What I will say is not anything for the company to increase, but maybe me and people who are really keen for the breastfeeding program… I would collaborate with the mothers and make forums and teach them breastfeeding (Theme 5, ST 5.4, Daycare #10).

Many daycare directors reported willingness to feed expressed milk to children under their supervision, provided they had appropriate refrigeration and bottles.

Most employers perceived they are providing sufficient support for lactating mothers through current maternity leave duration, scheduling flexibility and, in a few cases, on‐site daycare. Those who did not have on‐site daycares felt it could help support EBF: ‘having a baby care just within the premises…could help’ and is something ‘hotels would like to embrace’ (Theme 5, ST 5.5, Hotel #2). Some managers also felt lactation rooms would be beneficial, despite some concerns that they would go unused. One manager believed engagement with advocacy groups that help to advance the BF conversation could ensure the provision of these supports in the horticulture industry (Theme 5, ST 5.5, Farm #3).

Table 3 summarizes the interventions to improve EBF among employed mothers that were recommended by the different groups. The most cited supports were flexible work schedules, on‐site daycares, education and promotion regarding milk expression and increased maternity leave length. Mothers and alternate caregivers were more likely to suggest extending leave lengths and reducing employment hours following the return to work, whereas managers and healthcare providers considered strategies to enable BF at work.

**TABLE 3:** Recommended interventions to improve exclusive breastfeeding among working mothers

	Subtheme #	Mothers	Alternate caregivers	Healthcare providers	Daycare	Managers
1. Decrease hours or provide greater flexibility of hours upon return to work	5.1, 5.2	X	X	X	X	X
2. Provide on‐site daycare, allowing mothers to breastfeed during shifts	5.1, 5.3, 5.5	X		X	X	X
3. Increase the length of maternity leave	5.2	X	X		X	
4. Increase education and promotion of expression at perinatal appointments	5.3, 5.4	X		X	X	
5. Establish a lactation room at the workplace	5.1, 5.3, 5.5	X		X		X
6. Supply expression equipment, such as a pump, refrigerator and bottles.	5.1, 5.3, 5.4	X		X	X	
7. Educate daycare staff on expression and hygiene	5.3, 5.4			X	X	
8. Promote policy change and intervention at the industry and governmental levels	5.3, 5.5			X		X

**FIGURE 1:**
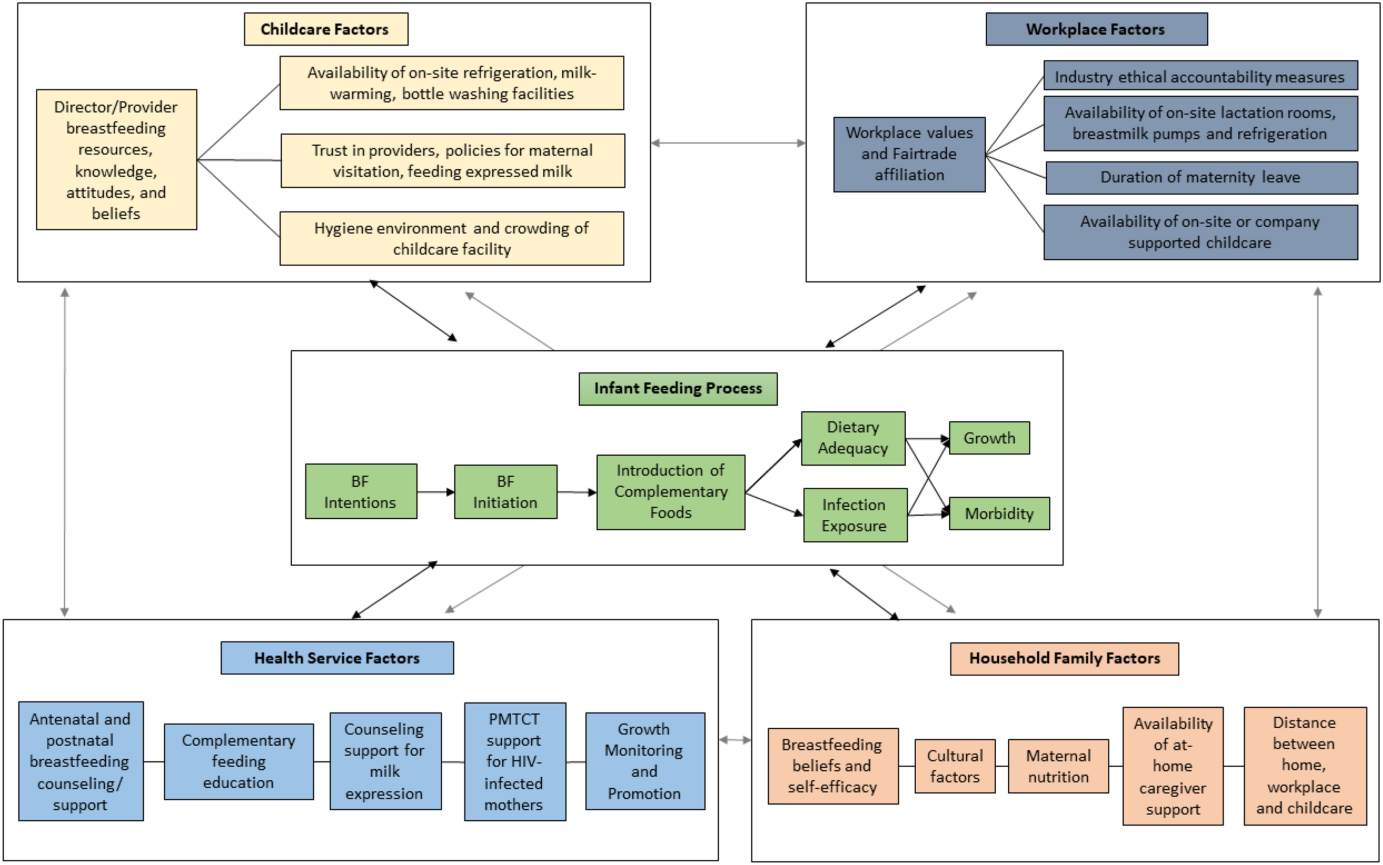
Conceptual framework of factors that influence breastfeeding practices among low wage, employed mothers. Childcare factors include the knowledge, attitudes and beliefs of the daycare directors and the environment, including the availability of milk expression supports, visitation and expression policies and hygiene. Health system factors include perinatal breastfeeding counselling/support, prevention of mother‐to‐child transmission support for HIV‐infected mothers and growth monitoring and promotion. At the household level, breastfeeding beliefs and self‐efficacy, culture, maternal nutrition, availability of at‐home childcare and distance to workplace influence breastfeeding practices. Finally, at the workplace level, organizational values and resources influence lactation support and subsidized childcare, on‐ or off‐site from the workplace

Figure 1 integrates the findings to illustrate the interconnected multilevel factors that influence infant feeding and EBF among employed mothers.

## 4 DISCUSSION

This is the first study to provide qualitative evidence about challenges to implementing workplace support for BF in Kenya following the 2017 Health Act. Our findings highlight an avid awareness of the recommended optimal duration of EBF among mothers and other stakeholders connected to the commercial agriculture and tourism industries. Despite strong recognition of ideal young infant feeding practices, these groups identified multiple employment‐related obstacles to EBF for the recommended 6‐month duration due to structural factors such as lack of access to lactation support at work and daycare and behavioural factors like the efficacy of expressing and storing milk or integrating BF within the work schedule. To enforce lactation provisions in the Health Act, the Government of Kenya proposed the Breastfeeding Mother's Bill that would impose a fine to companies with 50 or more employees who fail to establish a refrigerated lactation space refrigeration (Government of Kenya, 2019).

Our findings are consistent with other studies in LMICs that demonstrate that work outside the home is a barrier to EBF (Dearden et al., 2002) and that working mothers describe concerns over the feasibility and safety of BF at work (Horwood et al., 2020). An important contextual factor in this study context is long commuting distances to work that make the feasibility of visiting children at home or in community‐based childcare centres less feasible than in some contexts. Implementation of multipronged interventions can effectively improve BF practices in LMICs (Bosire et al., 2016; Chapman et al., 2010; Tylleskär et al., 2011). Despite the challenges to EBF faced by employed mothers, each participant group described potential EBF‐supportive interventions and policies across healthcare, community, and workplace settings to improve BF practices.

### 4.1 Flexible work schedules

Participants from each of the five categories cited that flexible work schedules and decreased hours upon returning to work would help mothers' ability to breastfeed exclusively for 6 months. While limited evidence from high‐income countries has not associated flexible schedules and lactation breaks with BF outcomes (Bai & Wunderlich, 2013; Jacknowitz, 2008), a recent review of drivers of EBF among employed women included data from seven LMICs and noted flexible hours as a facilitator of EBF but did not provide effect estimates (Gebrekidan et al., 2020). Studies guided by implementation science frameworks (Carroll et al., 2019) are needed to evaluate strategies to increase use of flexible schedules among working mothers in LMICs and to assess the impact of these interventions on EBF. In this setting, the benefit of flexible work on visits to children during shifts depends on access to affordable and efficient transportation to visit children. Therefore, interventions should consider local realities of commuting distances, transportation cost and time away from work.

### 4.2 On‐site daycare

Interviewees' second most common recommendation was on‐site daycare coupled with breaks for women to visit and breastfeed their children during the workday. Workplace daycares and permission to bring a child to work have been significantly associated with BF frequency and duration in various LMICs (Amin et al., 2011; Nkrumah, 2017; Osis et al., 2004), although evaluation on EBF duration or prevalence is lacking (Dinour & Szaro, 2017). The evidence base on employer‐based daycares' impact on BF practices is nascent but promising (Lundquist et al., 2019). Given that mothers travel long distances to their workplaces in the study context, we recommend expansion on on‐site daycare as a feasible strategy for increasing EBF and improving the overall quality of childcare.

### 4.3 Counselling

Participants also recommended increasing education on EBF for mothers and their communities, particularly on expressing breast milk. While health facility‐ and community‐based EBF counselling are the most common interventions delivered in LMICs (Olufunlayo et al., 2019), few studies have evaluated counselling or messaging to promote milk expression among working mothers (Ortiz et al., 2004; Valdés et al., 2000). Several culturally appropriate counselling aides have been developed and tailored to LMIC contexts (Global Health Media, 2020; UNICEF, 2019). Evaluation of these tools on milk expression practices remains an area for further investigation. Our findings suggest that mothers may benefit from counselling specific to milk expression as on‐site lactation rooms become available and milk expression is promoted.

### 4.4 Leave length

Unlike for above recommendations, there is already strong evidence on the association between maternity leave length and BF practices. Mothers, alternate caregivers and daycare directors suggested extending maternity leave beyond the current 14 weeks. Meta‐analysis data show for every month of paid maternity leave in LMIC, EBF prevalence increased by 5.9% points, BF duration lasted 2.2 months longer, and infant mortality decreased by 13% (Chai et al., 2018; Nandi et al., 2016). In Naivasha's floriculture industry, many mothers receive an additional month of paid time off though the addition of leave time to the mandated maternity leave. Additional extensions are likely to prove difficult for employers. In 2017, the Kenyan legislature voted down a bill to extend maternity leave to 6 months (Talbert et al., 2018), where business lobbyists argued that a longer leave was not economically sustainable (Wesangula, 2017).

### 4.5 On‐site lactation spaces

Workplace lactation rooms have been implemented and evaluated in a variety of contexts. Among factory workers, the provision of a workplace lactation space was associated with continued BF upon return to work, increased EBF duration and increased prevalence of EBF at 6 months (Basrowi et al., 2015; Chen et al., 2006; Rea et al., 1999). However, workplace managers in our study expressed some doubts regarding mothers' willingness to use such a facility, especially for expressing milk. Evaluations of maternal desire to pump and store expressed milk at workplaces are limited. Some documentation suggests negative maternal attitudes in Kenya (Talbert et al., 2018). Therefore, normalizing milk expression and storage and improving access to necessary equipment and supplies (e.g., pumps, storage bottles and handwashing stations) may be essential to realize workplace lactation spaces' potential. Demonstrations on expression from healthcare workers and especially from peers within the workplace context may prove beneficial to increase efficacy for breast milk expression.

The evidence base for workplace supports for EBF is moderate in high‐income countries, where numerous fair labour policies have been enacted (Abdulloeva & Eyler, 2013) but where many mothers still lack the necessary support from their employers (Taylor et al., 2020). Evaluations in HIC settings indicate that maternity leave (Mirkovic et al., 2014), flexible work schedules (Kimbro, 2006), private lactation spaces and provision of pumping equipment (Cohen et al., 1995) can improve BF practices. However, demographic characteristics and employment type may modify the impact of these supports (Taylor et al., 2020). Improving BF support in childcare is a noted priority in high‐income countries, where mothers desired help with selecting providers who can best facilitate BF goals and who view childcare as a paid‐for ‘service’ more than a supportive setting to meet their BF goals (Lundquist et al., 2019). Several of the most frequently desired supports for BF at work—on‐site daycare, flexible work schedules and counselling and supplies for breast milk expression—have not yet been well studied in LMICs. However, these recommendations reflect the top priorities of key stakeholders and highlight the need to develop and evaluate these initiatives. Taken collectively, the stakeholder recommended interventions hold great potential to improve BF practices among employed mothers. More evidence is needed to delineate their relative potential effects, and multipronged implementation is likely necessary to achieve substantial shifts in BF outcomes. One programme in Thailand demonstrated a significant 32% increase in EBF prevalence at 6 months after delivering a multicomponent intervention (Yimyam & Hanpa, 2014). Future evaluations should examine multiple, simultaneous workplace strategies to strengthen the evidence base and illuminate the most effective—and cost‐effective—components.

### 4.6 Strengths and limitations

The strength of our study is the large sample that included multiple perspectives from five participant groups. Together, these groups represent a range of influences on mothers' BF practices and reflect the multilevel effects on infant feeding (Rollins et al., 2016). Interviews were conducted in person and in the preferred language of the participant with fluent interviewers or translators. The inclusion of participants from two leading employment sectors within the study areas increases the generalizability.

There are several limitations to this study. First, the included population overly represented Fairtrade companies, which tend to be more employee‐centred and may be more likely to comply with BF legislation. The perspectives of farms not affiliated with Fairtrade, which may be less further along the implementation spectrum, were not well represented among the farm managers. Second, the study's data collection period occurred during the implementation of the 2017 Kenya Health Bill. Thus, many employers had not yet implemented lactation rooms at workplaces, limiting evidence on these supports. Third, we did not link mothers with their respective employers to validate responses regarding workplace support availability as the recruitment procedures for these two groups were independent of one another.

### 4.7 Policy implications

As Kenya's 2017 Health Act continues to be implemented, these findings can help guide the actions of policymakers and employers. Although the Health Act requires on‐site lactation rooms and flexible schedules to visit children daycares, our findings suggest that mothers may prefer visiting children in on‐site daycare rather than expressing and storing milk. Although milk expression and bottle feeding are important strategies to increase the consumption of human milk (Slusher et al., 2012), the additional steps of storing and feeding from a bottle may prove less feasible for mothers than visiting children at on‐site daycares to breastfeed directly. Further, pumping and storing milk may introduce opportunities for nutrient loss, microbial contamination and reduction of anti‐infective properties (Felice et al., 2017). Based on the BF Gear Model, research and evaluation can directly fuel advocacy, which, in turn, can change political will (Pérez‐Escamilla et al., 2012). These data contribute to the evidence base that influences advocacy for new policies, while also providing feedback and monitoring of the recently implemented legislation and policy cascade.

## 5 CONCLUSIONS AND RECOMMENDATIONS

Mothers employed in low‐wage work receive some supports from their employers for infant care responsibilities. Despite consistent knowledge of the child feeding recommendations and benefits of EBF, the need for mothers to return to work after maternity leave corresponds with numerous challenges. These include distance to childcare, inability to nurse during the workday and lack of support for and experience with milk expression, making EBF unattainable for most mothers in these industries. Participants' recommended interventions to support EBF for working mothers were consistent across participant groups; however, quality evaluation of some of these interventions is needed, especially in LMIC contexts. Improved intervention implementation, coupled with rigorous evaluation, is necessary to strengthen employed mothers' opportunities for optimal BF practices, including EBF, upon return to work.

## CONFLICTS OF INTEREST

The authors declare no conflicts of interest.

## CONTRIBUTIONS

SI, DD, JM, BS, CF, JW, LI and RN conceptualized the research study and contributed the methodology. SI, HS and HL collected the data. SI, HS, JK and HL analysed the data. SI and HS wrote the paper. All authors provided critical input into the review and revision of the manuscript.

## Supporting information

**Table S1**. Consolidated criteria for reporting qualitative studies (COREQ): 32‐item checklist
**Table S2**. Key themes and selected interview responses

## Data Availability

The data that support the findings of this study are available from the corresponding author upon reasonable request.
