# Supplementary material for: Exclusive breastfeeding among working mothers in Kenya: Perspectives from women, families and employers
Source: Matern Child Nutr. 2021 May 5;17(4):e13194. doi: 10.1111/mcn.13194 (PMC8476403; doi:10.1111/mcn.13194)
Supplement: Supplementary file 1 — Table S1. Consolidated criteria for reporting qualitative studies (COREQ): 32‐item checklist Table S2. Key themes and selected interview responses [file MCN-17-e13194-s001.docx]

**Supplemental Table 1**. Consolidated criteria for reporting qualitative studies (COREQ): 32-item checklist

Developed from: Tong A, Sainsbury P, Craig J. Consolidated criteria for reporting qualitative research (COREQ): a 32-item checklist for interviews and focus groups. International Journal for Quality in Health Care. 2007. Volume 19, Number 6; pp. 349-357.

| **Topic** | **Description** |
| --- | --- |
| **Domain 1: Research team and reflexivity** | |
| *Personal characteristics* | |
| 1. Interviewer/facilitator | Interviews were conducted by three investigators: XX an expert in maternal and child nutrition (2); XX, a Kenyan nurse fluent in Kiswahili; and (3): XX a student in global health and nutrition. |
| 2. Credentials | XX holds a PhD and is a senior researcher. XX holds a BS and XX. XX holds a BA. |
| 3. Occupation | Removed for review. |
| 4. Gender | Removed for review. |
| 5. Experience and training | Removed for review. |
| *Relationship with participants* | |
| 6. Relationship established | Participants were informed about the study aims before the interview, but did not interact with the interviewers before interviews. |
| 7. Participants knowledge of the interviewer | Participants received information that the interviews were being conducted by researchers. Participants interacted directly with the PI, who served as in-country study facilitator. The PI communicated with administrators at each hospital to receive approval for the study, enumerated study participants, followed up with interview participants, and managed participant reimbursement. The PI did not have previous knowledge of most interview participants prior to the beginning of the study. |
| 8. Interviewer characteristics | No characteristics were reported. |
| **Domain 2: Study design** | |
| *Theoretical framework* | |
| 9. Methodological orientation and theory | Semi-structured, in-depth, in-person interviews were conducted.  Grounded theory was used to guide the key informant interview process. |
| *Participant selection* | |
| 10. Sampling | We conducted 121 interviews across five participant groups. **Table 1** summarizes the respective sample sizes, interview locations, participation rates, and information sought from each group. Forty-two mothers employed in a flower farm or hotel, 20 fathers and alternate caregivers, 21 healthcare providers, 22 daycare directors, 16 farm, and hotel managers were interviewed |
| 11. Method of approach | Participants were contacted in person, via email and via phone to participate in the interview as described in the methods section. Interviews with farm and hotel managers and healthcare proviers were schedule. Participants were reimbursed with handsoap (valued at 400 KES) after the interviews.. |
| 12. Sample size | 119 interviews were completed. |
| 13. Non-participation | Fifty-eight survey participants did not respond or declined the request for interviews. In rare cases, a scheduled interview was not able to be completed due to issues with phone connectivity. Attempts were made via email and direct communication between CO, SP, and the participant to reschedule these interviews. |
| *Setting* | |
| 14. Setting of data collection | All interviews were conducted in person in Naivasha, Kenya. |
| 15. Presence of non-participants | Not applicable |
| 16. Description of sample | See Results section. |
| *Data collection* | |
| 17. Interview guide | The interview guides can be made available upon request to the authorship team. |
| 18. Repeat interviews | Repeat interviews were not conducted. |
| 19. Audio/visual recording | All interviews were audio-recorded. Recordings were transcribed verbatim and translated by XX. English interviews were transcribed by research assistant YY. |
| 20. Field notes | No field notes were collected. |
| 21. Duration | Interviews were 45-60 minutes, including administration of informed consent. |
| 22. Data saturation | Saturation was reached when no unique themes or responses arose during the interviews. |
| 23. Transcripts returned | Transcripts were not returned to interview participants for comment/correction. |
| **Domain 3: Analysis and findings** | |
| *Data analysis* | |
| 24. Number of data coders | Two; All transcripts were double coded by SP and SM using pre-specified codes. Discrepancies were first discussed between coders. Any outstanding coding differences were arbitrated through discussions with XX, who oversaw and reviewed the coding process, and AA, BB and CC, who served as a content experts. |
| 25. Description of the coding tree | The coding tree was based on domains identified in the survey: 1) discharge care, 2) follow-up care, 3) guidelines, 4) readmission, and 5) post-discharge mortality, and themes were identified based on this data. See Table 3. |
| 26. Derivation of themes | Data-derived themes were based on content analysis of double-coded verbatim transcripts. |
| 27. Software | Dedoose Version 8.0.35, web application for managing, analyzing, and presenting qualitative and mixed method research data (2018). Los Angeles, CA: Socio Cultural Research Consultants, LLC. |
| 28. Participant checking | Participants did not provide feedback on the findings. |
| *Reporting* | |
| 29. Quotation presented | See results section and Table 2, Box A, Figure 1 and Supplemental Table 2. |
| 30. Data and findings consistent | Yes; the data presented is representative of the findings. XX, YY and YY have extensive experience with pediatric care in Kenya and YY is a Kenyan trained pediatrician. They assisted with interpreting the findings in the Kenyan context. |
| 31. Clarity of major themes | Yes; see Table 2 and Supplemental Table 2. |
| 32. Clarity of minor themes | Yes; the research team collaborated to identify further understand divergent participant responses. Minor themes and divergent participant responses were analyzed during the interview analysis and presented in the manuscript. |

| **Supplemental Table 2**. Key themes and selected interview responses | |
| --- | --- |
| **Major Themes** | **Select interview Responses** |
| **Theme 1: Multiple participant groups identified formal employment as a challenge to practicing EBF for the recommended six-month duration.** | |
| **Sub-Theme #1.1: Despite recognizing the recommended six months duration for EBF and health benefits such as healthy growth and improved immunity, employed mothers reported beginning mixed feeding in preparation for their return to work after their three-month maternity leave.** | 1. "I breastfed my baby for a while, I think for 5 months before giving food. Then I began to feed him porridge, milk since I was trying to find a job because he had to get used to feeding porridge, bit by bit, as I would leave him when I get a job. He got used to taking porridge because if not, he could have because he could have cried a lot and disturb the caregiver” (Mother #31). 2. "For me I have to give food before I report back to work so that by the time, I go to work the baby will be used to feeding on other foods” (Mother #20). 3. "I started to feed by the end of maternity leave, the third month, I started him with milk and porridge lightly” (Mother #30). 4. "They would wish to exclusively breastfeed but they have to go back to work” (Daycare #9). |
| **Sub-Theme #1.2: Healthcare providers reported providing evidence-based breastfeeding education to mothers, but concurred that maternal employment is a challenge to practicing EBF for six months.** | 1. "They know because we are trying to teach them. Trying to educate them, but still when you educate them, they tell you 'Where will I get that time?' 'Cause they get up early, they go to work early, so no, they don’t have enough time to do exclusive breastfeeding” (Healthcare #18). 2. "Reason number one [mothers face challenges to EBF] is the working hours. Usually they come from their houses around six…from six to six-thirty am and usually get into their house by five or six [at night]. So, most women are not able to breastfeed exclusively because of number one, the timing” (Healthcare #2). 3. "Mostly those mothers fail to do exclusive breastfeeding because of work, and especially those who are working in the flower farms who go very early in the morning and coming in the evening. They are not able to breastfeed during the day” (Healthcare #8). 4. "Even if she needs to breastfeed exclusively, at times, no, she has to go and work so she to meet her household needs” (Healthcare #13). 5. "I hear one is supposed to breastfeed for six months before weaning, although for me I cannot manage because of work” (Mother #20). |
| **Theme 2: Though managers and healthcare providers recognized pumped milk as an opportunity for working mothers to continue to practice EBF, few employed mothers reported expressing breastmilk.** | |
| **Sub-Theme # 2.1: Managers, healthcare workers, and daycare directors all concurred that milk expression might be challenging for or undesirable to mothers. They voiced doubts that mothers would readily adapt to expressing and storing milk.** | 1. "But I think [expressing milk] is still not, it hasn’t gotten to African mothers expressing milk and all that...Culturally it is not being very acceptable apart from what now the ones who have been a little bit modernized. Local mothers I don’t think would be, have not seen that” (Hotel #1). 2. "Most of them have not been trained on how to do the pumping of the breast because if you don’t have the part they feel ashamed that the milk would be coming out and that smell of breastmilk they don’t want. That is why most of them don’t take fluids and they don’t have the psychological aware that they have small kids and they are worried people might shame them” (Healthcare #15). 3. "Stigma. Stigma is a big problem. So, they don’t believe in feeding the baby the [expressed] milk because they have never heard of such a thing” (Healthcare #14). 4. "[Mothers] don’t have enough education. Most of them they don’t understand about expressing milk. They think most expressing milk only works for premature babies when they are in the hospital. But the moment the mother has left the hospital, she stops breastfeeding” (Healthcare #15). |
| **Sub-Theme 2.2: Most mothers had heard of milk expression and many mothers expressed an interest in pumping to extend EBF after returning to work. However, they did not feel expression was feasible due inadequate instruction and demonstration, lack of hygienic environment, and equipment (i.e., breast pumps, bottles and refrigeration).** | 1. “I heard that you can express and keep it in the fridge, but I have never heard that other people like me do who don’t have a fridge [can do it]” (Mother #16). 2. "One, they don’t have the facilities even for expressing the milk. They have no interest; they don’t know” (Daycare #2).smae  3. "No they don’t because most of them are living in villages, they are vulnerable and they don’t have that machine to express the milk, and in fact we don’t talk about how to do it because we don’t know” (Daycare #1). 4. "I have heard about [pumping milk], but I can’t do it because I don’t have the items to pump. I hear you must keep the milk in a clean place, and also the person feeding the baby must check the hygiene” (Mother #31). 5. “No [I would not breastfeed my baby in the middle of the day], you know there are so many chemicals at the farm” (Mother #32). 6. "I can’t pump my milk considering I don’t have a safe place to store the breastmilk, and also the utensils used to store the breastmilk, I fear them because of the hygiene of the person I going to leave the baby with” (Mother #30). |
| **Sub-Theme #2.3: Daycare directors suggested that mothers are hesitant to leave expressed milk to feed their children during the workday because they lack refrigeration at their daycares to store milk.** | 1. "[EBF] has not been very successful. Some of them, one of them was not very satisfied, but it is because we don’t have the facilities. I know that we would need a fridge for that baby. The milk can continually be fresh and so we don’t have that. We don’t have power. So, we are limited. We would love to help and I know what we need and hopefully God will help us in future. In such a way that all the mothers here can breastfeed, can feed their children by breastmilk” (Daycare #2).  2. "Because all the day cares in Nairobi that I know cannot afford to keep refrigerators. But normally [mothers] must understand that the milk which is being expressed normally stays in a clean place for less than 8 hours and it is okay, so it doesn’t need to be refrigerated. So long as the day care is well ventilated and the place where they are keeping the milk is clean, then the milk is okay. Ventilation of rooms is the most important part. We don’t encourage refrigerators because there is more risk to use them” (Healthcare #15). 3. "I want to know how to keep and feed the expressed breastmilk if a mom left them” (Daycare #14). 4. "If I had a fridge [breastfeeding mothers] will accept [it], but still expressing milk is a challenge so they opt to feed cow’s milk” (Daycare #21). 5. "I have seen them; there is a trend. Immediately when [breastfeeding mothers] go back to work, they really try...The first day they will bring like three quarters of a bottle of expressed breastmilk, and then they go to work. They stay in hot conditions, they don’t feed, and they have all of the stress of working. And by the end of that week the mother will not provide even a little amount of milk. So, they just stop. But they try the first week” (Daycare #3). |
| **Sub-Theme 2.4: Lack of trust in providers negatively influences mothers' willingness to leave expressed milk at daycares.** | 1. "After expressing, where you store that milk, and besides the person you leave the baby with, you are not sure they will keep the milk safe. You see nowadays you can't trust people much as opposed to the old days. They can decide to do other things with that milk. Let’s say if I decide to take the baby to the daycare with that milk, the person has too many other children. Do you think they will fully take care of that milk?” (Mother #20). 2. "They usually take their children to a child care unit that is not registered. Most of them they are not registered. Just somebody who decided to put a baby care unit there, a baby center for where mothers bring children, and they have no, it is not registered, so even the care is not up to date. Then because you cannot leave the breastmilk to any person, so they prefer to buy the cow’s milk, and that’s a challenge to the children, the babies” (Healthcare #17). 3."According to me it is hard because even if I leave the milk, I am not sure he will be fed the same milk or not be fed the same milk because I will not be there to see” (Mother #6). 4. "For those working mothers, they are never sure how the person left with the baby is feeding the baby. For example, I had to stop working so as to take care of our child when the mother is not at home. I did not want the child to be taken to baby care. I have visited so many daycares and the state there is not good” (Father #4). |
| **Theme 3: Healthcare providers reported that though they provide evidence-based breastfeeding education consistent with global guidance on infant feeding and HIV, employed mothers are challenged to practice EBF through six months.** | |
| **Sub-Theme #3.1: HIV-infected mothers were often enrolled in HIV care services, which included education on infant feeding. Despite these additional supports, providers observed that work poses a challenge for some mothers to practice EBF through six months.** | 1. "For the mother who is HIV positive, because the baby is at risk of getting the infection, we do tell them if they are going to practice the exclusive breastfeeding, they should do it continuously and not give the baby any other food stuff” (Healthcare #12). 2. "If a mother is HIV positive, there are chances that the baby can get HIV so we give them options. The mom can decide to breastfeed or not to breastfeed. The chances of the baby getting the virus are there, although they are very remote. So they make their own informed choice after teaching. But most of them, about 90% or more, chose to breastfeed for the benefits. Because we have some interventions reporting that we can prevent the transmission from the mother to the baby. So, women like to breastfeed. Number one for the health of the baby, and then the bond of the mother and the baby (Healthcare #14)." 3. "HIV positive mothers are more careful” (Healthcare #3). 4. "Initially, they were very resistant. There was the perception that HIV positive mothers should not breastfeed their babies. But nowadays, they are very comfortable with it. They take it positively, and they follow the rules that we are giving them. Exclusive breastfeeding is a must. But at some point, we get challenges, like a mother says she only has three months maternity leave so after three months she should be going back to work so how will the baby be managed? So, we tell her she should pump, she should express the milk, and tell her that breastmilk takes eight hours before. But most of them don’t take it positively because they say they don’t have enough milk, and how will the caregiver handle the baby and clean... and maybe there is still fear about the stigma” (Healthcare #7). |
| **Sub-Theme 3.2: Some HIV-infected mothers reported additional benefits due to their status, such as extended maternity leaves and transportation to return home during the workday to breastfeed. Other HIV-infected mothers indicated that they could only manage to practice EBF through three months when the return to work would force them to begin mixed feeding or wean from mother’s milk if EBF was no longer an option.** | 1. "We find that [HIV positive mothers] have already introduced the baby to breastfeeding, and the same time they are giving the baby the porridge because they cannot, the baby cannot survive with only the breastmilk from morning to evening waiting for the mothers to come back from work” (Healthcare #12). 2. "The HIV positive women are the ones doing exclusive...because they are also supported. Sometimes when they go to their clinics, they are given some free financial…so they can be able to do exclusive breastfeeding” (Healthcare #2). 3. "Because they are being counseled on how to breastfeed, many of them they do follow the advisories that we give them, but sometimes they do get a mother and maybe she is working the flower farm and she only gives three months exclusive breastfeeding and after three months they are given a break for three months and then after three months they are supposed to go back to work and they don’t have anyone to leave the baby with so maybe they leave it with a neighbor or with a child care and those people that are taking care of the babies they starting to feed them some porridge or some other things and that’s how mostly we see the baby turning positive” (Healthcare #5).  4.“Most mothers don’t [express milk], but one who was HIV positive and it was only one case but she had the kid at home and the milk was there and once he is 6 or 7 months then they stop breastfeeding because it is exclusive then she brought him here. So we have not had a case where parents have to express milk” (Daycare #10).  5. Moderator: When you go back [to work], will you be able to breastfeed as [you are] now?  Respondent: No, I will have to stop breastfeeding completely.  Moderator: Why?  Respondent: Because of my HIV status (Mother #16). |
| **Theme 4: Workplaces recognized the need to support breastfeeding and identify some practical employee benefits; however, employers describe maternal-level limitations to using workplace benefits.** | |
| **Sub-Theme #4.1: Most farm managers reported that mothers prefer to arrive later or leave earlier rather than visiting children during the workday for feeding.** | 1. "I think that the challenge [of nursing in the middle of the day] was basically the aspect of them going and coming back is an issue. We have special places near, but much easier cause they would go and come but here we don’t have special places. So it was very convenient for them to go and breastfeed and come back. So, it was also hard for them, so in essence, there was also that because some of them normally take their children to baby care, so they preferred the morning hours so that the morning they have their babies so they are ready to breastfeed them so then they are ready to take them and then in the afternoon they normally leave at the same time as the others. So, majority of them prefer early time because it was giving them a lot of challenge” (Farm #5). 2. "For going home during the day, it would likely be too far and too expensive” (Hotel #4). 3. "Some of them will not go to breastfeed, but maybe go to collect their child or go to the house or something like that. But ideally that slot is given to them. They are allowed to leave early and things like that" (Farm #3).  4. "To the level of my knowledge and I think to the best of my ability, what we are doing is perfect because it is very flexible for us. It is not like it is restricted for seven hours. That they have to work for seven hours. We can…I mean we have the arrangements of those who probably want to work late, work normal hours, or they come…they work up to…they combine their lunch hour with their tea break, so then they leave much, much earlier. Because we give one hour for lunch, forty minutes for break, so when they report at around 7:30am, some leave at midday. So then that gives them another shorter period to take care of their children. We also have those arrangements where probably a mother comes and probably has issues with the baby, we take it very personally. We don’t deny them all of those off. We don’t deny them time off, but still paid because at least we understand that they are going to take care of their children” (Farm #1). 5. "We can also give what we call a ‘broken shift.’ Broken shifts mean that because most of the people usually reside not very far from the hotel, maybe in Karagita, you know, in Naivasha town... Broken shift means that you can work for four, or just … three hours, then you take one hour, go home, feed the kid, and then you can come back” (Hotel #2). |
| **Sub-Theme #4.2: Few farms or hotels reported having designated lactation areas at the time of the study, and several workplace managers described plans to comply with new national policy.** | 1. "Because you know the hygiene of handling everything would be a little bit more complicated for the workers and you need more education. And so, we thought we couldn’t really add that much, but at least for the office ladies there is a higher level of understanding and hygiene can be better, then we have provided that for them” (Farm #4). 2. "Although the government has also come up with a legislation that says you should have a space for breastfeeding children, it’s a new law. It’s a very new law that we haven’t been able to implement this so far. But this legislation was just passed by parliament a few months ago, two to three months ago, that we should create a space where breastfeeding mothers can feed their children” (Farm #2). 3. "This year a new labor law came active and you have to have a breastfeeding room and we … said to ourselves, 'Are we just building a room, and we put a signboard on the door, and that is it?’ Or, are we actually looking into ‘How can we help to make it into a success?' One of the things we are looking into now is if we could get a hospital-grade pumping with removable cups and a fridge that we can actually have it here so our employees can actually use it. Maybe five people at the same time, because at the moment I think we will have five breastfeeding ladies at the same time” (Farm #6). |
| **Sub-Theme #4.3: Managers described that cultural factors may prevent mothers from using private lactation rooms at workplaces once established. Healthcare providers also noted that mothers may lack knowledge of and experience with expressing and storing milk.** | 1. "If I put [a lactation room] here, then nobody is going to use it because it is not something that is accepted yet. I don’t know how it is in Nairobi and in the higher educated sectors, but I think that will be the biggest challenge to actually having people to understand the right thing to do” (Farm #8). 2. "That mother who is there, who never went to school, she just came from the village and she knows the mother is supposed to breastfeed the milk, there is no way I am going to tell her, 'Remove this milk, keep it in a container, I will give it to the child during the day.' It doesn’t come. Because she grew up and she never saw it being done. She never had any school and so there is nobody who told her the milk should be removed... It can be very complex when you try to convince someone who does not understand why it should be done so, who does not have the facility to do so, who does not trust what I am doing is right. When you don’t trust what you are doing is right, there is no way out. She’ll tell you, 'Let me keep my milk. I will give it in the evening.' So then that becomes the whole process. That makes the whole process a cultural thing” (Farm #1). 3. "I think [a lactation room] is something that will finally be introduced, but I know that it will also receive a little bit of resistance because there are those people who do not believe in removing the milk and storing. They believe that baby should have the milk right from the source. So, to change someone’s mindset out of that, it requires a lot of time” (Farm #1).  4. "I think also we have to address the cultural issues. Yeah, there are some cultures that find it odd these kinds of things, and you cannot force them. There are some cultures that if you express milk it is against their tradition of behaviors and such things” (Farm #5). |
| **Theme 5: Despite significant challenges, opportunities to improve support for EBF exist in household, community, workplace, and health system settings.** | |
| **Sub-Theme #5.1: Though mothers offered various suggestions to improve the feasibility of continuing EBF, including flexible hours and lactation support, many preferred the addition of workplace daycare.** | **Provision of on-site day care** 1. "I would like for them to at least to build us a small place where we can breastfeed our babies at a certain time, and be taken care of while we work... I would [build a day care at work] because it would save time for the others and also there would be more attachment between the baby and the mother. Also, it would save the mother from all the motorbike expenses if your home is far” (Mother #23). 2. "What I would request is there was a baby care at the farm so we can take our children there so whenever we are needed to breastfeed or be given breaks to breastfeed during the day. That will be of great help other that the one hour they give. Because getting transport home takes a while, there is not much difference with the other employees” (Mother #37).  **Decreased or more flexible hours upon return to work** 3. "I would give them permission to go breastfeed during lunch hour and they come back. [If they don't have any fare to go], the company bus is there to take them and bring them back” (Mother #34).  **Lactation room and workplace supports** 4. "If I had my own bottles and, for example, a pump I would just express at the bathrooms [at work]; no one will be bothered...I will be very happy [if work provided a room and a fridge] because it will be benefitting my baby” (Mother #37). |
| **Sub-Theme #5.2: Fathers and alternate caregivers described several changes that would improve the feasibility of EBF for working mothers. The most frequent suggestion from this group was a more substantial reduction and flexibility of work hours upon returning to work.** | **Increased length of maternity leave** 1. “The maternity leave for the working moms is only 3 months. If they could extend it to six months, it would be better so that the child would have that appropriate time to really breastfeed” (Father #1).  **Decreased hours upon return to work** 2. "Some companies may say we gave three-month maternity leave. But three months… it's not six months, but the mother must continue breastfeeding after six months. It could be a sacrifice to provide that room or time for the mother to breastfeed at least three times a day, and she should go to work at least one hour late and that one hour and 30-minute lunch break to travel. If they are from far, at least provide a room. It should not be looked at as a disadvantage of one hour, but as an advantage of building a next employee in the future and if you don't do it, you will risk having an employee who is always sick” (Theme 5, ST 5.2, Father #7).  **Providing extra nutrition**  3. "I work hard for the mother to get enough nutritious food for her to produce breastmilk for the baby, also for the baby too I work hard for her to have enough to eat so as for her to be strong" (Father #5). 4. "I buy milk and I also buy her food she needs like porridge...In order for her to have enough breastmilk to breastfeed” (Father #3). |
| **Sub-Theme #5.3 Healthcare providers most frequently report improvement of perinatal and community education to support the continuation of EBF, but also recommend on-site daycares, supplies to support milk expression, and governmental policy changes to maternity leave and lactation rooms.** | **Expression Support** 1. "I think one thing we need to be able to encourage mothers first of all and allow them to understand that if you can produce milk even when you go back to work there are ways you can do it. Secondly, just encouraging them on and just explain to them about storage. I think that’s the greatest challenge right now. And so maybe having handouts showing you know this is how long milk can stay in room temperature, this is how you can express. I think those really help. Visual aids for moms will really help just to show them and things they can carry home to show them that it’s actually possible. And just to have testimonies around, even in a group session have a mother say, ‘I did it for 6 months and I went back to work’” (Healthcare #18).  **Education** 2. "Maybe we need to empower the caregivers to go through the workshops and trainings on exclusive breastfeeding and maybe all that entails the importance of breastfeeding...we could create a breastfeeding area where we do the demonstrations for those mothers with the concerns and maybe can be done, maybe once in a week or after a period of time” (Healthcare #4). 3. "Education is power. These mothers need to be educated. If you tell them that breastmilk takes 8 hours for it to get spoiled, they will understand and they will practice it. And telling them that doing exclusive breastfeeding will boost the immunity of this child, they will do it... When they have not yet delivered, they should be educated. In the community, we have the CHW, the Community Health Workers, they too do their volunteering work, they should be educating the mothers. When the mother delivers here, we always give health education and we insist on breastfeeding, that exclusive breastfeeding. I think that’s how it should be” (Healthcare #7). 4. "Those who stay at home also have poor knowledge about children. So, we can link with the community to also do health messages—like churches or other schools because we also have students who can learn about breastfeeding and also link with other managers from other farms so we can see how we can help these mothers continue breastfeeding and continue work at the same time” (Healthcare #3). |
| **Sub-Theme #5.4: Daycare directors were in consensus that mothers need both education and the provision of supplies such as pumps, bottles, and refrigerators to support breastmilk expression as a means to facilitate EBF upon return to work.** | **Education** **1**. "Getting more education is important because If I am educated, I will have facts to tell the mothers and they will be willing to listen. For example, some used to say pampers are not good for boys so when they ask you don’t have any reason to explain to them. If you teach us more about exclusive [breastfeeding], then we will teach them with facts” (Daycare #21). 2. "There needs to be some training of some sort because most mothers, though they are aware, they have no interest. It’s not something they really understand well...If the hospitals would provide that it would really help because people believe doctors a lot. And nurses, so if they would go to take their children to the clinic and they find a service, a health service provider who is able to train them, talk to them about it, I believe it would work. And then maybe still enforce it from the day cares” (Daycare #2). 3. "What I will say is not really anything for the company to increase, but maybe me and people who are really keen for the breastfeeding program to come in and educate the mommas and let them know because as I told you earlier, most of them don’t really care...so I think what I would do is time to time to corroborate with the mothers and make forums and teach them breastfeeding” (Daycare #10).  **Expression support** 4. "If we have the equipment we can [feed expressed milk]. Yes, we can, for the health of the babies” (Daycare #1). 5. "If I had that fridge, I would tell them to express and leave it with me to feed the kids” (Daycare #20). |
| **Sub-Theme #5.5: Most employers perceived they are providing enough support for lactating mothers through the current maternity leave duration, flexibility in hours, and, in a few cases, on-site daycare. Those who did not have on-site daycares felt it could be helpful in supporting EBF.** | **On-site day care** 1. "It is important that [farms] have these crèches. Or they have lactation areas. But, I wouldn’t speak for others, but for us maybe more sensitization conversations about it so we are more deliberate about it and deliberate about the message we pass about breastfeeding, what are the effects, what are the stories around it, and maybe collect stories from employees who have exclusively breastfed, and perhaps use those stories to encourage their peers” F 2. "Having a baby care just within the premises I think it could help...just walking few distance to feed your kid. You can just go and check with your kid; I think that is also something that maybe a hotel would like to embrace” (Hotel #2).  **Policy Change** 3. "For the horticulture district, so those I am sure if you go through them they would be able to bring the people because I know the Kenya Private Association, Kenya Private Sector Association, KPSA, had this drive for breastfeeding bill and I think they are part…one of the reasons the health bill, which there now is the breastfeeding bill moved so far. So, I think I have seen, particularly for the horticulture industry would help in advancing that conversation. One, they are an advocacy firm, but they are also a standard. They also have their standards, so they can be able to maybe implement that...So it could be a bit difficult for [other farms] to take ownership of the breastfeeding position. But I think when the people know the needs, the importance, then the people will follow through themselves. You don’t need anyone to push it for them. So, I think it is really more sensitization could add more value” (Farm #3).  **Lactation room** 4. "I think the idea of having a place for nursing mothers to breastfeed their children would be really, really good and great. Just to ensure that children are breastfed until the time they are six-months" (Farm #2). |
